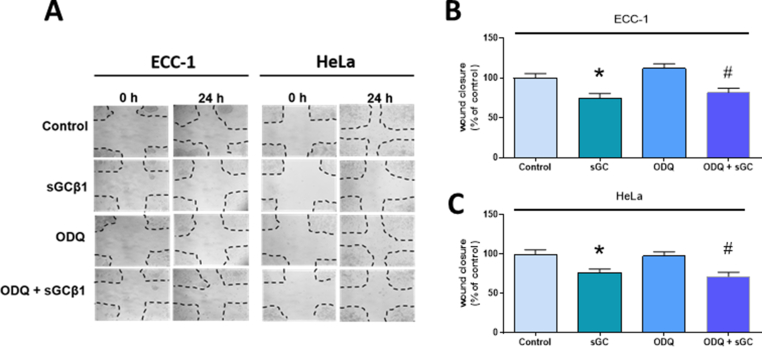# Soluble guanylyl cyclase beta1 subunit targets epithelial-to-mesenchymal transition and downregulates Akt pathway in human endometrial and cervical cancer cells

**DOI:** 10.1016/j.heliyon.2023.e23927

**Published:** 2023-12-19

**Authors:** Lucas H. Acosta, María Teresa L. Pino, María Victoria Rocca, Jimena P. Cabilla

**Affiliations:** CONICET-Universidad Abierta Interamericana. Centro de Altos Estudios en Ciencias Humanas y de la Salud. Buenos Aires, Argentina

**Keywords:** Soluble guanylyl cyclase β1 subunit, Cell death, Cell migration, Endometrial cancer, Cervical cancer, Cell signaling

## Abstract

Endometrial and cervical cancer are among the most frequently diagnosed malignancies globally. Nitric oxide receptor-soluble guanylyl cyclase (sGC) is a heterodimeric enzyme composed of two subunits, α1 and β1. Previously we showed that sGCα1 subunit promotes cell survival, proliferation, and migration, but the role of sGCβ1 subunit has not been addressed. The aim of the present work was to study the impact of sGCβ1 restoration in proliferation, survival, migration, and cell signaling in endometrial and cervical cancer cells. We found that sGCβ1 transcript levels are reduced in endometrial and cervical tumors vs normal tissues. We confirmed nuclear enrichment of sGCβ1, unlike sGCα1. Overexpression of sGCβ1 reduced cell viability and augmented apoptotic index. Cell migration and invasion were also negatively affected. All these sGCβ1-driven effects were independent of sGC enzymatic activity. sGCβ1 reduced the expression of epithelial-to-mesenchymal transition factors such as N-cadherin and β-catenin and increased the expression of E-cadherin. sGCβ1 impacted signaling in endometrial and cervical cancer cells through significant downregulation of Akt pathway affecting some of its main targets such as GSK-3β and c-Raf. Our results show for the first time that sGCβ1 exerts several antiproliferative actions in ECC-1 and HeLa cell lines by targeting key regulatory pathways.

## 1 Introduction

Nitric oxide receptor-soluble guanylyl cyclase (E.C. 4.6.1.2) is an enzyme constituted by two subunits, α and β, α1 and β1 being the most frequently expressed in tissues. The two subunits are encoded by different genes under independent regulation [1,2]. The heterodimer is ubiquitously present in all human tissues [3,4]. This enzyme acts as the main receptor and effector of nitric oxide (NO), which in turn catalyzes the synthesis of 3′, 5′-cyclic guanosine monophosphate (cGMP) derived from guanosine 5′-triphosphate. The NO transduction signal is crucial in animals and plants [5].

Previously we showed that 17β-estradiol (E2) differentially affects sGCα1 and sGCβ1 expression in endocrine tissues by increasing sGCα1 expression and decreasing or not affecting sGCβ1 expression, depending on exposure time [[6], [7], [8], [9]]. The role of these subunits in processes unrelated with classical cGMP formation has been investigated only in recent years. The role of sGCα1 in prostate cancer cell progression was reported first by Cai et al. [10,11], followed by our investigation demonstrating the participation of sGCα1 in endometrial and cervical cancer cell proliferation and migration [12]. Soon after, sGCα1 expression levels were shown to correlate with tumor progression in breast cancer biopsies from patients [13,14]. Altogether this evidence points to sGCα1 as an important factor promoting tumor proliferation and migration.

In contrast, the sGCβ1 role in cGMP-independent functions has been much less investigated. sGCβ1 was shown to associate with chromosomes during mitosis in glial cells thereby impeding cell division and promoting cell cycle arrest [15]. sGCβ1 gene promoter region was also found to be hyperacetylated and hypermethylated in breast cancer cells, and restoration of sGCβ1 subunit expression decreases cell proliferation and migration [16,17]. In malignant tumors, sGCβ1 protein expression was lower than that of benign and normal breast tissues [14]. Meta-analysis from breast cancer datasets from patients showed that lower sGCβ1 expression is related with a worse prognosis [17].

Endometrial carcinoma (EC) is the fourth most frequent cancer in women and its incidence is currently growing worldwide. Estrogen exposure (mainly E2) is considered the highest risk factor in EC onset and progression [18]. High-risk human papillomaviruses (HPV)-mediated cervical cancer (CC) is the fourth most often diagnosed female cancer globally. While E2 has been generally unrelated to CC, there is increasing though still contradictory evidence linking E2 with CC aetiology, onset, and progression [19].

Previously, acute E2 administration was found associated with diminished sGCβ1 expression in uterus [20] and chronic E2 or E2-like compounds administration tended to decrease sGCβ1 protein levels [9]. Also, underscoring the hypothesis that sGCα1 and sGCβ1 play different roles in cancer cell biology, we demonstrated that E2 and E2-like compounds upregulate sGCα1 expression in uterus [9] and derived tumor cell lines and that sGCα1 is an important factor in cervical and endometrial cancer cell proliferation, survival, and migration [12].

Akt pathway plays a key role in controlling survival and apoptosis [21] and is constitutively active in many malignancies, including endometrial [22] and cervical carcinomas [23]. PI3K/Akt pathway as a therapeutic target has been increasingly studied and multiple clinical trials are currently exploring treatments for endometrial cancer [24] and cervical cancer [25]. Fully active PKB/Akt mediates many cellular functions comprising angiogenesis, cell proliferation, cell survival, growth, and apoptosis, among others. PDK-1 is a PIP3-dependent upstream activator of Akt. PTEN is a tumor suppressor involved in a broad range of human cancers, acting as a major negative regulator of the PI3K/Akt signaling pathway [26]. Glycogen synthase kinase-3β (GSK3β) plays a crucial role as a downstream component of the PI3K/Akt cell survival pathway whose activity can be hindered by Akt-driven phosphorylation at Ser9 [27]. c-Raf belongs to Ras/Raf/MEK pathway, which is inactivated by Akt through phosphorylation at Ser289, constituting a key crosstalk point between PI3k/Akt and Ras/Raf/MEK/ERK pathways [28].

In the present work we aimed to study the effect of sGCβ1 independent of its enzymatic function on cell proliferation, migration, and invasion in endometrial and cervical tumor cell lines by augmenting its expression through an adenoviral vector. Here we reported that restoration of sGCβ1 expression in human endometrial and cervical cancer cells lacking sGCβ1 reduced cell viability and migration in a cGMP-independent fashion. sGCβ1 was found in the nucleus and cytoplasm and significantly impacted on key hallmarks as epithelial-to-mesenchymal transition and activation of the Akt pathway. The findings from the present investigation contribute to improve our comprehension of the function of sGCβ1 in gynecological malignancies.

## 2 Materials and methods

### 2.1 Bioinformatic analysis

Signature-based statistics for normal/cancer comparison were collected by GEPIA2 (Gene Expression Profiling Interactive Analysis), the web server for large-scale analysis of cancer-related genomic datasets, available at http://gepia2.cancer-pku.cn [29]. GEPIA2 is a highly referred resource for analyzing RNA sequencing expression data of 9736 tumors and 8587 normal samples from the TCGA and the GTEx databases. Tumor/normal differential expression analysis was performed.

### 2.2 Cell cultures

ECC-1 is a hormone-responsive human endometrial epithelial cancer cell line. HeLa is an HPV-infected human cervical cancer cell line. ECC-1 and HeLa cell lines were obtained from ATCC (Manassas, VA) and generously donated by Laboratorio de Inmunología de la Reproducción (IByME-CONICET), and by Dr. Viviana Blank (IQUIFIB, Facultad de Farmacia y Bioquímica, Universidad de Buenos Aires), respectively. Cells were maintained in Roswell Park Memorial Institute (RPMI) media (Gibco, Waltham, MA, USA) containing 10 % fetal bovine serum (Internegocios, Buenos Aires, Argentina) and penicillin-streptomycin mixture (50 units/mL and 50 μg/mL). Cells were kept at 37 °C and 5 % CO_2_. All experiments were conducted in RPMI supplemented with 5 % fetal bovine serum (RPMI-S-FBS). Control of each experiment was carried out using the same culture media.

### 2.3 Adenovirus

Viral vector containing sGCβ1 or sGCα1 sequences were kindly gifted by Dr. Andreas Papapetropoulos [30]. Adenoviral vectors were amplified by Dr. Eduardo Cafferata (Instituto de Investigaciones Bioquímicas de Buenos Aires, CONICET). Stocks of 4.78 × 10^12^ PFU/mL and 1.78 × 10^13^ PFU/mL were obtained for sGCβ1 and sGCα1, respectively. ECC-1 and HeLa cells were seeded on 6- or 24-well plates with a minimum of 80 % confluence and infected with adenovirus containing sGCβ1 sequence (Av-sGCβ1-GFP), sGCα1(Av-sGCα1-myc), empty vector or vector containing GFP alone (Av-GFP), with a multiplicity of infection (MOI) varying from 0 to 100 in serum-free media for 4 h at 37 °C. After this period, media were discarded and replaced by RPMI-S-FBS. Experiments were performed up to 48 h after infection. Since no significant differences were found between empty vector and Av-GFP in cell viability, cell cycle distribution, or nuclear morphology (Supplementary materials Fig. S1), empty vector was chosen as control for the following experiments.

### 2.4 Cell viability

MTT (3-(4,5-dimethylthiazol-2-yl)-2,5-diphenyl-2*H*-tetrazolium bromide) assay was used to quantify mitochondrial activity as an indirect indicator of surviving/proliferating cells. Cells (2x10^4^) were seeded in 96-well culture plates and transfected with different MOI of Av-sGCβ1-GFP, Av-GFP or empty vector. Cells were incubated with complete medium for 48 h. Then, cells were incubated with 10 μL of a 2 mg/mL MTT solution in PBS for 1 h at 37 °C. The medium was discarded and 100 μL 0.01 N HCl in isopropanol was added to each well to resuspend formazan. Optical density at 595 nm was measured in a 96-well plate reader (Glomax, Promega). For enzymatic sGC inhibition experiments, the cells were incubated with 0.1 % DMSO (dimethyl sulfoxide; vehicle control) or 1 μM ODQ (1H- [1,2,4]oxadiazolo [4,3-a]quinoxalin-1-one) after virus transfection up to 48 h. This concentration was selected based on literature [31] to ensure sGC activity inhibition without affecting other haem-containing proteins [32,33].

### 2.5 Cell cycle analysis by flow cytometry

After treatment, cells were scraped off with a rubber policeman, and fixed with an ice-cold solution of 70 % ethanol in PBS. After centrifugation, pellets were resuspended and incubated for 30 min at 37 °C in 0.2 mL of staining solution containing 50 μg/mL propidium iodide (PI) in PBS with 0.2 mg/mL of DNase-free RNase A. DNA content was measured by flow cytometry with a Becton Dickinson FACScalibur flow cytometer (San Jose, CA, USA). Analysis of cell cycle distribution was performed with WinMDI 2.8 software (http://facs.scripps.edu).

### 2.6 Nuclear morphology and immunocytochemistry

Cells were seeded onto 24-well glass coverslips. After treatment, cells were incubated for 30 min at 4 °C in a solution containing 4 % formaldehyde in PBS. Nuclei were permeabilized for 15 min at room temperature with 6 N HCl in 1 % Triton X-100 in PBS followed by neutralization with 0.1 M sodium borate in 1 % Triton X-100 in PBS for 15 min at room temperature. Non-specific binding sites were blocked with 5 % normal serum in 0.2 % Triton X-100 for 2 h at room temperature. Cells were incubated overnight at 4 °C with anti sGCα1 primary antibody (1:500) and the secondary antibody conjugated to Alexa fluor 488 (1:250) after washing with 0.5 % Triton X-100 in PBS. Cells overexpressing sGCβ1-GFP were visualized directly. Cells were mounted in VectaShield anti-fade solution containing Hoechst 33258 for nuclear staining. For the evaluation of sGCβ1 and sGCα1 localization, cells were observed under a Zeiss LSM 510 Meta laser scanning confocal microscope using a 40X oil-immersion 1.2 numerical aperture objective and BP420-490 and BP505-530 filters (Carl Zeiss, Germany) from the Microscopy and Bioimaging Facility at the Leloir Institute Foundation (Buenos Aires, Argentina). For nuclear morphology, cells were photographed with a Flexacam camera coupled to a Leica DM750 fluorescence microscope (Leica Microsystems, Austria). Apoptotic and mitotic indices were calculated as: number of apoptotic or mitotic nuclei/total number of nuclei × 100.

### 2.7 Scratch wound assay

7x10^4^ cells were plated in a 24-well plate and infected with Av-sGCβ1 or empty vector (control). The monolayer was wounded with a pipette tip and floating cells were removed by washing with PBS. Images of the scratched area were taken at 0 and 24 h and evaluated by ImageJ software (National Institutes of Health, USA). Wound closure was calculated as (area 0 h-area 24 h)/area 0 h and expressed as percentage of control.

### 2.8 Transwell migration assay

Cell migration was carried out in an 8 μm-pore size Boyden chamber (BD Biosciences, San José, CA, USA). 200 μL of a suspension of control and Ad-sGCβ1-infected cells (2.5x10^5^ cells/mL in serum-free media) were seeded onto the upper chamber of each of the transwell inserts. RPMI with 10 % FBS was added to the bottom chamber as a chemoattractant. After 24 h, the non-migrated cells were detached from the upper chamber with a moistened cotton swab. The cells that had migrated through the membrane were fixed with ice-cold methanol, stained with Giemsa, photographed, and counted under a light microscope.

### 2.9 Gelatin zymography

Protein content from each conditioned medium was measured by Bradford and gel was loaded with adjusted volumes to reach an equal amount of protein (80 μg/lane). Aliquots (30–40 μL) of cell-conditioned media from control or Av-sGCβ1-infected cells were mixed with Laemmli modified buffer composed of 10 mM Tris–HCl (pH 6.8), 2 % SDS, 0.03 % bromophenol blue, and 10 % glycerol. Samples were resolved in a 6–7.5 % SDS-PAGE containing 1 % gelatin. To remove SDS, gels were washed twice for 30 min with 50 mM Tris–HCl buffer (pH 7.4), 2.5 % Triton X-100, and three times for 5 min with 50 mM Tris–HCl buffer (pH 7.4). Gels were incubated in reaction buffer containing 50 mM Tris–HCl, 0.15 M NaCl, and 10 mM CaCl_2_ (pH 7.4) for 24, 48, or 72 h at 37 °C and stained with 0.5 % Coomassie brilliant blue R-250. Clear bands resulting from MMP-2 activity were photographed and quantified by Gel-Pro Analyzer 3.1. Values were normalized to β-actin immunoreactive bands from each sample.

### 2.10 Western blot analysis

About 40–60 μg of total protein from each sample were boiled for 5 min in Laemmli sample buffer, resolved on 10–12 % SDS–PAGE, and transferred to polyvinylidene difluoride membranes. Non-specific binding sites were blocked for 24 h at 4 °C using 5 % bovine serum albumin in 1 % T-TBS (blocking buffer). Membranes were incubated overnight at 4 °C with primary antibodies in blocking buffer and 2 h at room temperature with the corresponding HRP-conjugated secondary antibody. Membranes were exposed to Biolumina ECL detection kit (Kalium, Buenos Aires, Argentina) and chemiluminescent images were acquired using Genetools 4.3.14 software in a G:Box Chemi XRQ gel documentation system (Syngene, Cambridge, UK). The intensity of the bands was quantified using GelPro Analyzer 3.0 software (Media Cybernetics, MD, USA). All antibodies used are listed in Supplementary Materials Table S2.

### 2.11 Statistical analysis

Data were graphed as mean ± SE and assessed through either Student's ‘*t*’ test or one-way analysis of variance (ANOVA) followed by Tukey's or Dunnett's test. Statistical significance was defined as P-values less than 0.05. All statistical analyses were performed using GraphPad Prism 8.00 for Windows GraphPad Software (San Diego, CA, USA). At least three independent replications of each experiment were conducted.

## 3 Results

sGCβ1 is downregulated in endometrial and cervical carcinomas. sGCβ1 localizes in cytoplasm and nuclei of ECC-1 and HeLa cells.

We first analyzed the data of GEPIA2 database based on the mRNA sequencing output from cervical squamous cell carcinoma and endocervical adenocarcinoma and uterine corpus endometrial carcinoma. We found that sGCβ1 transcript levels were significantly reduced in all these cancers compared to their respective normal adjacent tissues (Fig. 1**A**).

**Fig. 1:**
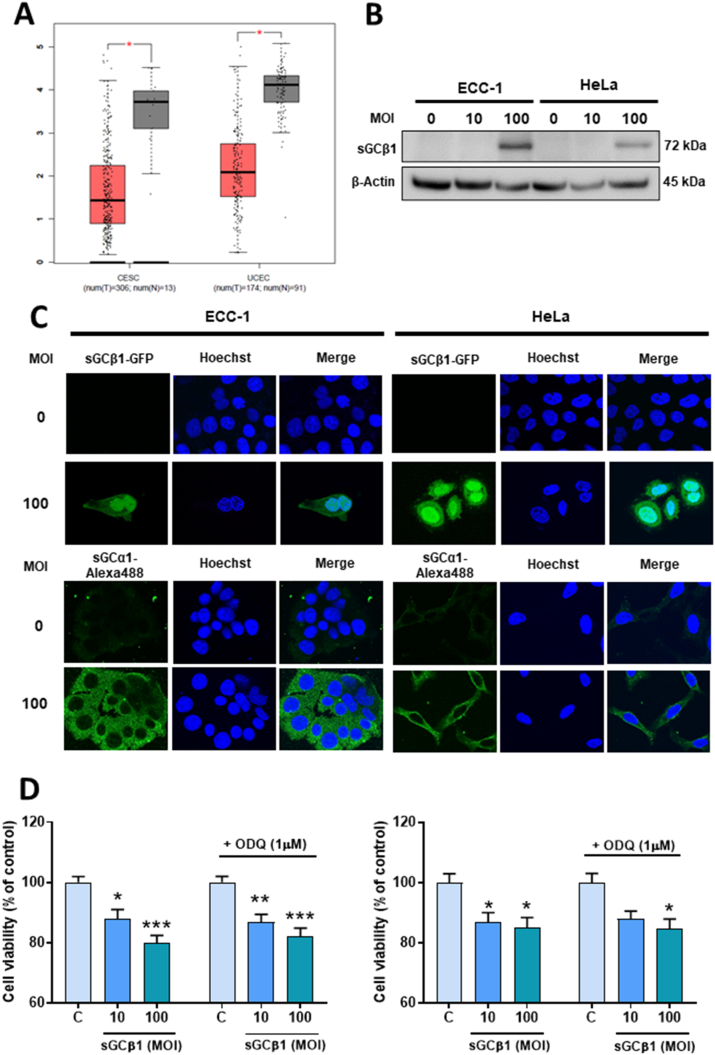
**SGCβ1 expression levels in endometrial and cervical cancer and subcellular localization of sGCβ1. (A)** Boxplot graphs of GUCY1B1 expression in uterus corpus endometrial carcinoma (UCEC), cervical squamous cell carcinoma, and endocervical carcinoma (CESC) by comparing paired normal (grey boxes) and tumor tissues (red boxes) from RNA-seq data at GEPIA2. **(B)** sGCβ1 protein levels after overexpression in ECC-1 and HeLa cells. Full, non-adjusted blot images are provided in Supplementary Materials S4. **(C)** Localization of sGCβ1-GFP in ECC-1 and HeLa cells (green, upper panels) and immunostaining of sGCα1-FITC (lower panels) visualized by confocal microscopy. Blue represents nuclei stained by Hoechst 33258. Representative images obtained at 40 × . **(D)** Cell viability of ECC-1 (left) and HeLa (right) determined after 48 h by MTT assay. ANOVA followed by Dunnett's test, *p < 0.05, **p < 0.01, ***p < 0.001 vs respective control (N = 5). (For interpretation of the references to colour in this figure legend, the reader is referred to the Web version of this article.)

To study the role of sGCβ1 in these cellular models, we first determined basal expression levels of sGCβ1. ECC-1 and HeLa cells showed nearly undetectable sGCβ1 protein levels. sGCβ1 expression was only detected at MOI 100 (Fig. 1**B**). Therefore, this MOI was chosen for subsequent experiments. Next, we sought to determine sGCβ1 subcellular localization. We found that, after sGCβ1 overexpression, the sGCβ1-GFP marker was present in cytoplasm as well as in nucleus (Fig. 1**C**). In order to rule out a technique-based artifact, we overexpressed sGCα1 subunit through a similar adenoviral vector containing full-length sGCα1 sequence [30]. sGCα1-overexpressing cells showed an exclusively cytoplasmic immunoreactive marker concordant with cytosolic sGCα1 expression (Fig. 1**C**). All together, these results showed that sGCβ1 expression was decreased in endometrial and cervical cancers and that sGCβ1 localized in both cytoplasm and nucleus.

### 3.1 sGCβ1 overexpression decreased cell viability, augmented subG0/G1 DNA content and apoptotic index

**Fig. 2:**
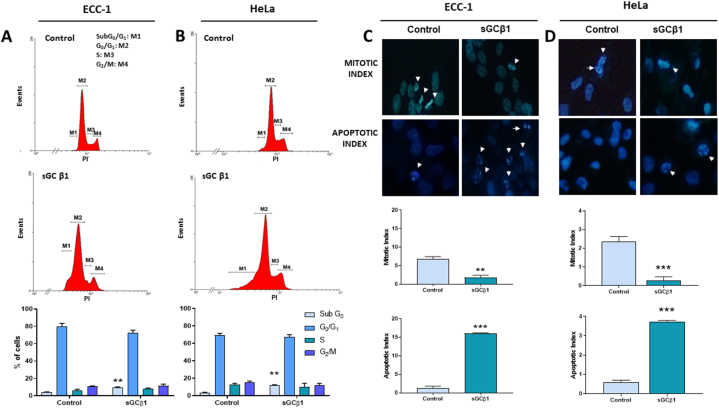
**SGCβ1 overexpression reduced mitosis and increased apoptosis.** Cells were infected with Av-sGCβ1 or empty virus (control). sGCβ1-transduced or control cells were stained with PI and analyzed by flow cytometry. Representative histograms (upper panel) and quantification of cell cycle distribution (lower panel) of **(A)** ECC-1 cells and **(B)** HeLa cells. Nuclear morphology was studied by Hoechst 33258 stain. Representative images obtained at 40 × (upper panel). Bars represent the mean ± SE of mitotic or apoptotic indices, expressed as percentages of total cell number (lower panel) of **(C)** ECC-1 cells and **(D)** HeLa cells. ANOVA followed by Dunnett's test, **p < 0.01, ***p < 0.001 vs respective control (N = 3).

Next, we studied the impact of sGCβ1 overexpression on cell viability. In both cell lines, sGCβ1 significantly reduced cell viability after 48 h, measured by MTT assay (Fig. 1**D**). This effect was not dependent on cGMP formation since treatment with a sGC inhibitor (1 μM ODQ) did not modify cell viability. The cell cycle distribution of PI-stained cells was examined to validate these findings. As expected, in both cell lines, sGCβ1 overexpression was associated with a significant rise in the percentage of subG0/G1 DNA content after 48 h, whereas no significant changes were observed in G1, S and G2/M cell cycle stages (Fig. 2**A** and **B**). Furthermore, nuclear morphology was studied to confirm these results. sGCβ1 overexpression significantly increased apoptotic index and decreased mitotic index in both cell lines (Fig. 2**C** and **D**).

### 3.2 sGCβ1 overexpression decreased cell migration and downregulated MMP-2 activity

**Fig. 3:**
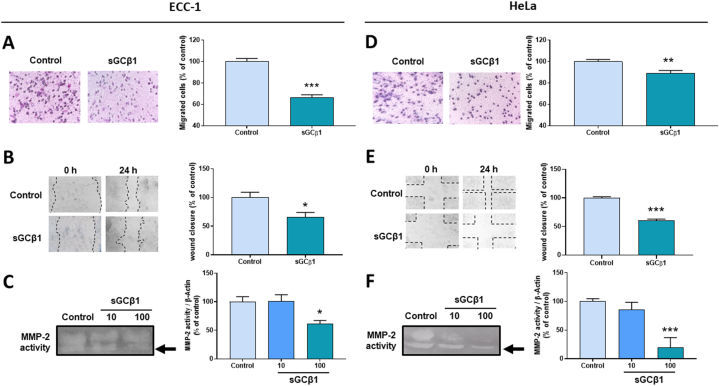
**SGCβ1 overexpression decreased cell migration and MMP-2 activity.** Cells were infected with Av-sGCβ1 or empty virus (control) at increasing MOIs. Cell migration was evaluated by **(A,D)** transwell assay and **(B,E)** wound healing assay. Left panels: representative images. Bars represent the mean ± SE of migrated cells or wound closure area after 24 h incubation, as percentage of control. **(C,F)** MMP-2 protein activity. Conditioned media were run in SDS-PAGE containing 1 % gelatin. Bars represent mean ± SE of average densitometric values of clear bands after 48 h normalized with β-actin content from each treatment. Full, non-adjusted gel images are provided in Supplementary Materials S4. ANOVA followed by Dunnett's test or Student's *t*-test, *p < 0.05, **p < 0.01, ***p < 0.001 vs control (N = 3).

Tumor cell migration from primary tumor to neighboring tissues or distant organs is one of the hallmarks of cancer. To assess whether sGCβ1 could also affect cell migration, we ran scratch motility and transwell migration assays. sGCβ1 overexpression significantly reduced the migratory capacity of both, ECC-1 and HeLa cells after 24 h of treatment (Fig. 3**A** and **B** and **D,E**). This effect was shown to be independent of sGC enzymatic activity since 1 μM ODQ failed to impede sGCβ1's inhibitory effect on cell migration (Supplementary Materials Fig. S3). These results suggest that sGCβ1 by itself not only affects cell fate but is also involved in cell migration.

Matrix metalloproteinases (MMPs) are a group of zinc-dependent endopeptidases. These secreted proteins are able to cleave extracellular matrix proteins, playing an important role in the pathogenesis of different diseases included but not limited to inflammation, tumor growth, and cancer metastasis [34]. Of the MMPs, MMP-2 acts as a key enzyme that has been linked to tumor metastasis and physiologic function in endometrial cancer [35] and to lower overall survival in cervical cancer [36]. Next, we addressed whether sGCβ1 could affect MMP-2 activity in ECC-1 and HeLa cells. We found that, after sGCβ1 overexpression, MMP-2 activity was significantly decreased in both cell lines (Fig. 3**C** and **F**). These results tally with with the sGCβ1-driven decrease in migration.

### 3.3 sGCβ1 overexpression decreased epithelial-to-mesenchymal transition markers

Epithelial-to-mesenchymal transition (EMT) is a key program in tumorigenesis. One EMT hallmark is the upregulation of N-cadherin followed by downregulation of E-cadherin which facilitates cancer invasion and metastasis. Considering that sGCβ1 caused a reduction in cell migration (Fig. 3), we explored whether sGCβ1 affected the expression of some EMT markers in both cell lines.

**Fig. 4:**
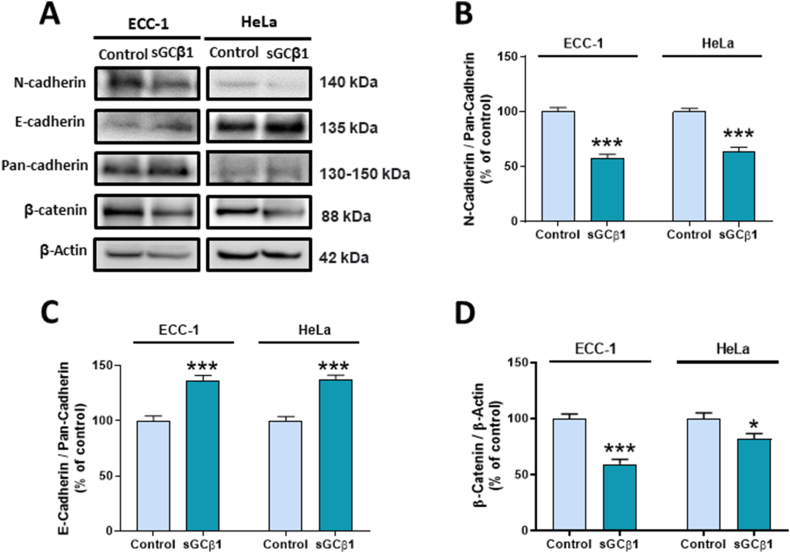
**SGCβ1 modified epithelial-to-mesenchymal transition marker expression. (A)** A representative Western blot of N-cadherin, E-cadherin, pan-cadherin and β-catenin on sGCβ1-overexpressing cells. Full, non-adjusted blot images are provided in Supplementary Materials S4. **(B**–**D)** Bars represent mean ± SE of average densitometric values of N-cadherin, E-cadherin, and β-catenin compared to pan-cadherin and β-actin, respectively. Student's *t*-test, *p < 0.05, ***p < 0.001 vs control (N = 3).

sGCβ1 was shown to significantly decrease N-cadherin and β-catenin protein expression (Fig. 4**A**, **B**, and **D**), whereas E-cadherin protein levels were significantly upregulated in both, ECC-1 and HeLa cells (Fig. 4**A** and **C**). These results suggest that sGCβ1 negatively regulates cell motility and migration and may explain the inhibition of cell migration seen in transwell and scratch motility assays.

### 3.4 sGCβ1 overexpression reduced the activation of Akt pathway

The Akt pathway is vital for cell survival, mediates many cellular functions including EMT, and is constitutively active in various cancers like endometrial and cervical carcinomas [22,23].

**Fig. 5:**
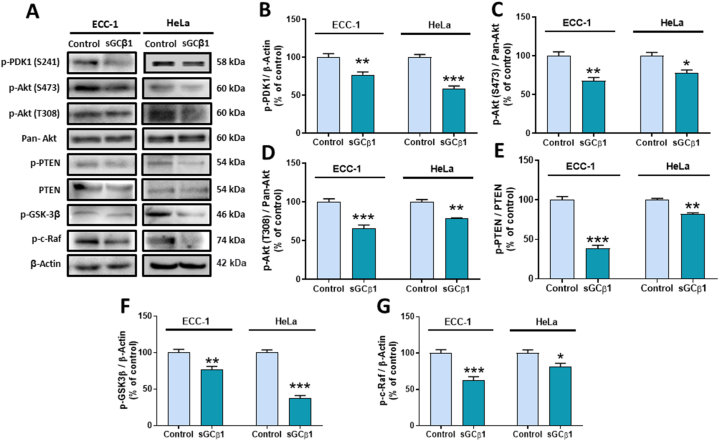
**SGCβ1 downregulated Akt signaling pathway in endometrial and cervical cancer cells. (A)** A representative Western blot of *p*-PDK1, *p*-Akt (S473), *p*-Akt (T308), pan-Akt, *p*-PTEN, PTEN, *p*-GSK-3β (S9) and p-*c*-Raf on sGCβ1-overexpressing cells. Full, non-adjusted blot images are provided in Supplementary Materials S4. **(B**–**G)** Bars represent mean ± SE of average densitometric values of *p*-PDK1, *p*-Akt (S473), *p*-Akt (T308), *p*-PTEN, *p*-GSK-3β, and p-*c*-Raf compared to pan-Akt, PTEN and β-actin. Student's *t*-test, *p < 0.05, **p < 0.01, ***p < 0.001 (N = 3–5).

We addressed whether Akt pathway is affected by sGCβ1 overexpression. sGCβ1 restoration reduced PDK1 activation (Fig. 5**A** and **B**). In line with this finding, phosphorylation of Akt at S473 and T308 was significantly decreased, indicating a decline of fully activated Akt (Fig. 5**A**, **C**, and **D**). As expected, inhibitory phosphorylation levels of Akt targets: PTEN, GSK-3β, and c-Raf were in turn also diminished, thereby confirming sGCβ1-driven Akt pathway inhibition (Fig. 5**A**, **E**, **F**, and **G**). These results suggest that sGCβ1-mediated antitumoral effects are mediated, at least in part, by Akt pathway downregulation.

## 4 Discussion

The dual-nature role of NO signaling pathway including sGC in cancer pathogenesis is not conclusive and varies radically among tissues. Many pro-tumor and anti-tumor effects of this pathway are attributed in part to sGC activity [37] although the participation of cGMP-independent mechanisms has been suggested [38]. Only a few reports have studied the role of each sGC subunit individually in cancer.

Our analysis showed here that sGCβ1 mRNA expression was decreased in endometrial and cervical tumors compared to normal adjacent tissues. cGMP/PKG pathway has also been found to be downregulated in endometrial cancer [39] and sGCβ1 expression was reported to be reduced in breast cancer cells [13,14,17]. All this evidence points to a potential role of sGCβ1 in tumor biology.

We found that sGCβ1 decreased cell viability and increased cell death, compatible with apoptosis, as seen in nuclear morphology. The percentage of cell death (15–20 %) was similar to that reported for sGCβ1-overexpressing glioblastoma cells after 48 h [40]. Since adenoviral vectors promote transient protein expression, we are currently developing an expression system to assess the long-term effects of sGCβ1 restoration on cell viability. Here we demonstrated that the reduction in cell viability was independent of cGMP production, since sGC enzymatic activity inhibition by ODQ did not avoid the effects observed after sGCβ1 overexpression in the current model. Previous evidence obtained in a glioblastoma model supports our findings [15,40]. Another study reported that restoration of sGC activity decreased cell growth and viability in breast cancer tumor cell lines. Noteworthy: this study showed that sGCβ1 mRNA and protein levels were far above those of sGCα1 subunit after demethylation; therefore, it is likely that sGCβ1 itself could contribute to those effects [16].

Confocal microscopy revealed that sGCα1 is located in cytoplasm, whereas sGCβ1 exhibited cytosolic as well as nuclear localization for both cell types. This finding concords with results reported on glia [15], differentiating embryonic cells [41] and U87 glioblastoma cells [40]. All this evidence strengthens the concept that sGCα1 [[10], [11], [12]] and sGCβ1 subunits have independent roles beyond forming a heterodimeric enzyme. Nuclear localization of sGCβ1 has been associated with physical interaction with chromosomes impeding mitosis [15] and, more recently, with an increase in p53 transcription [40]. In our experimental models, sGCβ1 binding to p53 promoter could explain in theory the effects observed in ECC-1 cells; however, the results obtained in p53-deficient HeLa cells [42] suggest the participation of p53-independent mechanisms. More studies are needed to fully elucidate the nuclear effects of sGCβ1.

We have shown that sGCβ1 restoration decreased cell migration, as seen in wound healing and transwell assays. The experiments were performed after 24 h of sGCβ1 overexpression, a time lapse when cell death is still negligible. Supporting this evidence, a relation between sGCβ1 and metastasis was described in melanoma cells where sGCβ1 expression loss was related to a highly metastatic phenotype, in contrast to melanocytes and nonmetastatic melanoma cells that expressed normal sGCβ1 levels [43]. To our knowledge, this is the only evidence that relates sGCβ1 with cell migration and metastasis. MMP-2 is one of the most important enzymes involved in cell migration, invasion, and tumor progression in endometrial and cervical carcinomas [35,36]. Furthermore, it is considered a predictive factor of worse prognosis in endometrial carcinoma [44]. Here we demonstrated that sGCβ1 restoration reduced MMP-2 activity from cell supernatants. Acquisition of migratory properties and invasiveness is tightly associated with EMT. E-cadherin is a transmembrane cell adhesion protein that is considered a predictor of better prognosis in endometrial and cervical cancer [45,46]. In contrast, augmented N-cadherin expression is linked to an increased migratory and invasive phenotype [47]. Here we report that sGCβ1 restoration increased E-cadherin protein levels together with a reduction of N-cadherin protein expression, which explains, at least in part, the sGCβ1-induced reduction in cell migration. All together, these findings suggest that sGCβ1 favors a more differentiated, less invasive tumor cell phenotype.

β-catenin participates in cell-cell adhesion and signal transduction, playing a critical role in the Wnt/β-catenin pathway involved in cell survival, cell proliferation, and EMT [48]. GSK-3β phosphorylates β-catenin leading to its ubiquitination-dependent proteolysis. In the present study, sGCβ1 restoration reduced total β-catenin protein levels, which could negatively affect Wnt pathway. This hypothesis is currently being investigated.

Bearing in mind that Akt pathway is a central node of many signaling pathways including EMT [49,50], we studied whether sGCβ1 restoration could impact on this signaling pathway. sGCβ1-overexpressing cells showed a downregulation of Akt pathway evidenced by a reduced phosphorylation state of PDK-1, Akt, and several of its downstream targets such as PTEN, GSK-3β, and c-Raf. Supporting our findings, it was reported that PI3K/Akt pathway activation downregulates sGCβ1 expression in vascular smooth muscle [51]. Akt hypophosphorylation decreases GSK-3β inhibition which could ultimately explain GSK-3β-driven β-catenin degradation.

GSK-3β is also involved in cell cycle control by phosphorylation-mediated degradation of cyclin D1 and E [52]. sGCβ1-driven GSK-3β hypophoshorylation is likely related to the observed decrease in cell survival and proliferation in the current experimental model. Similarly, the anti-apoptotic role of Akt has been widely reported [53]. In our experiments, sGCβ1-driven Akt downregulation could be responsible to some extent for the increase in cell apoptotic indices in ECC-1 and HeLa cells. More experiments are needed to confirm this hypothesis.

E-cadherin expression is mostly regulated by transcriptional and post-translational mechanisms as well as protein turnover [54]. Akt pathway has also been shown to promote EMT by activating transcription factors such as Snail and Twist, ultimately affecting E-cadherin expression. Inhibition of Akt in oral cancer cells led to an increase of E-cadherin [55]. In our study, sGCβ1-dependent E-cadherin upregulation could also be explained at least in part by Akt downregulation. The detailed mechanisms by which sGCβ1 targets Akt and EMT need to be clarified.

**Fig. 6:**
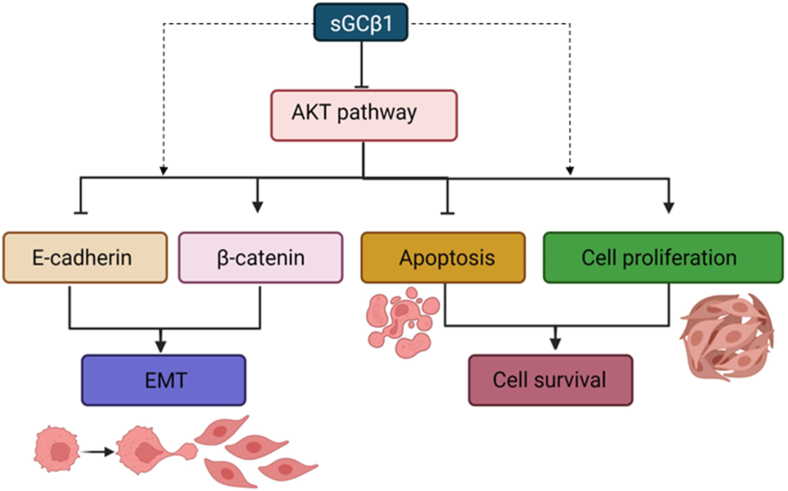
This proposed model shows how sGCβ1 decreased cell viability and migration by targeting Akt signaling pathway in endometrial and cervical cancer. The potential participation of other sGCβ1-driven mechanisms is depicted with dashed lines. Figure created with BioRender.com.

In sum, we have shown here for the first time that sGCβ1 subunit decreased tumor cell viability and migratory capability by targeting two critical points such as EMT and Akt pathway, leading to a less aggressive tumor phenotype in endometrial and cervical cancer cells (Fig. 6). All these findings may help us to understand the low sGCβ1 expression found in endometrial and cervical tumors. This in vitro approach indicates that sGCβ1 should be recognized as a potential therapeutic target for future studies aimed at decreasing gynecological tumor aggressiveness.

## Data availability statement

Data associated with this study has not been deposited in a publicly available repository. Data will be made available on request.

## CRediT authorship contribution statement

**Lucas H. Acosta:** Writing – review & editing, Writing – original draft, Investigation, Formal analysis, Data curation. **María Teresa L. Pino:** Writing – review & editing, Writing – original draft, Investigation, Formal analysis, Data curation. **María Victoria Rocca:** Writing – review & editing, Investigation. **Jimena P. Cabilla:** Writing – review & editing, Writing – original draft, Supervision, Project administration, Funding acquisition, Formal analysis, Conceptualization.

## Declaration of competing interest

The authors declare that they have no known competing financial interests or personal relationships that could have appeared to influence the work reported in this paper.

## Figures and Tables

**figs1:**
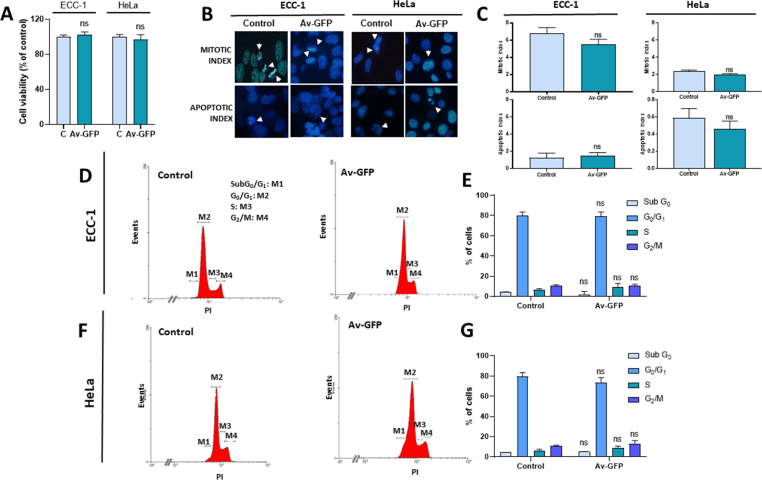


**figs2:**