# Supplementary material for: Soluble guanylyl cyclase beta1 subunit targets epithelial-to-mesenchymal transition and downregulates Akt pathway in human endometrial and cervical cancer cells
Source: Heliyon. 2023 Dec 19;10(1):e23927. doi: 10.1016/j.heliyon.2023.e23927 (PMC10777080; doi:10.1016/j.heliyon.2023.e23927)
Supplement: Multimedia component 1 [file mmc1.docx]

**Table S2.** Antibodies used for Western blotting and immunostaining.

| **Antibody** | **Company** | **Catalog number** | **Dilution** |
| --- | --- | --- | --- |
| sGCβ1 | Sigma | G4405 | 1:1000 |
| sGCα1 | Cayman Chemical | 160895 | 1:1000 |
| β-actin | Cell Signaling | 4970 | 1:1000 |
| N-cadherin | Cell Signaling | 13116 | 1:1000 |
| E-cadherin | Cell Signaling | 3195 | 1:1000 |
| β-catenin | Cell Signaling | 8480 | 1:1000 |
| Pan-cadherin | Cell Signaling | 4073 | 1:1000 |
| p-PTEN (S380) | Cell Signaling | 9551 | 1:1000 |
| PTEN | Santa Cruz | sc-7974 | 1:500 |
| p-Akt (S473) | Cell Signaling | 4060 | 1:1000 |
| p-Akt (T308) | Cell Signaling | 13038 | 1:1000 |
| Akt (pan) | Cell Signaling | 4691 | 1:1000 |
| p-GSK-3β (S9) | Cell Signaling | 5558 | 1:1000 |
| p-PDK1(S241) | Cell Signaling | 3438 | 1:1000 |
| p-c-Raf (S259) | Cell Signaling | 9421 | 1:1000 |
| Goat anti-rabbit Alexa 488 | Thermofisher | A1108 | 1:250 |
| Goat anti-rabbit IgG-HRP | Jackson | 111-035-003 | 1:2000 |
| Goat anti-rabbit IgG-HRP | Cell Signaling | 4074 | 1:2000 |
| Anti-mouse IgG-HRP | Dako | P0447 | 1:2000 |
