# Supplementary material for: Soluble guanylyl cyclase beta1 subunit targets epithelial-to-mesenchymal transition and downregulates Akt pathway in human endometrial and cervical cancer cells
Source: Heliyon. 2023 Dec 19;10(1):e23927. doi: 10.1016/j.heliyon.2023.e23927 (PMC10777080; doi:10.1016/j.heliyon.2023.e23927)
Supplement: Multimedia component 2 [file mmc2.docx]

S4. **Full gel and blot images from Figures 1, 3, 4, and 5.**

**Figure 1.**

Antibody anti-sGCβ1

Original chemiluminescent image and converted to gray scale


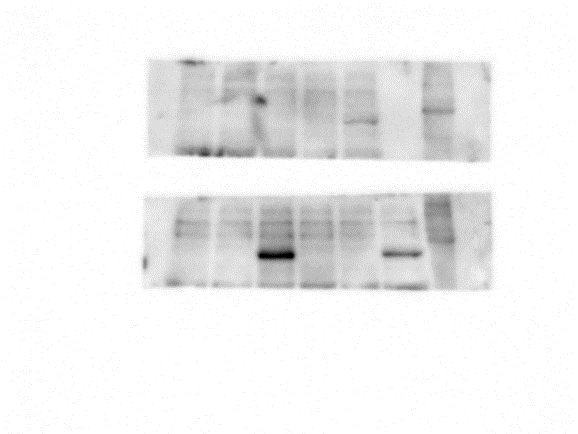


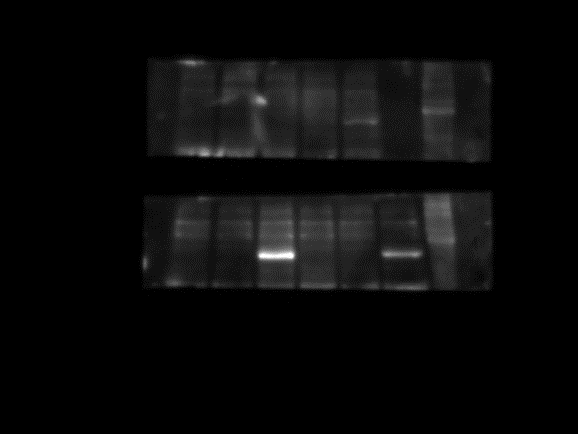


Selected bands

Antibody anti-β-actin

Original chemiluminescent image and converted to gray scale


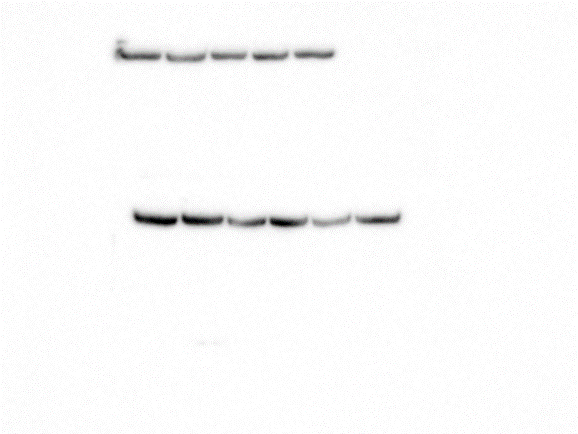


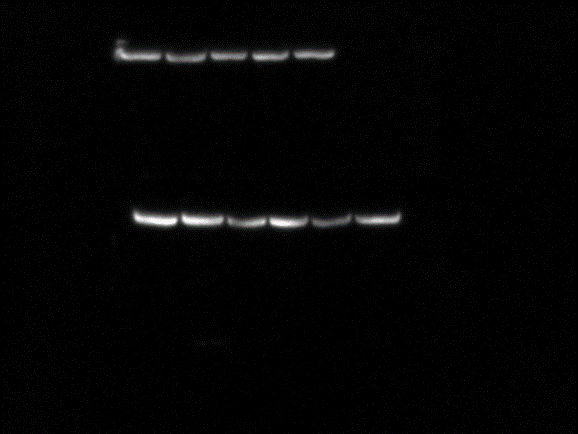


Selected bands

**Figure 3.**

**HeLa**

Original image and converted to gray scale

**
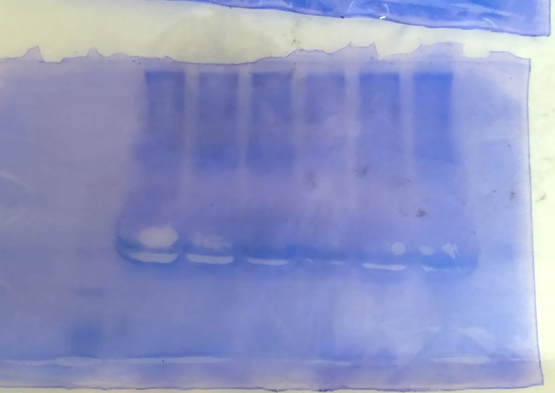
**


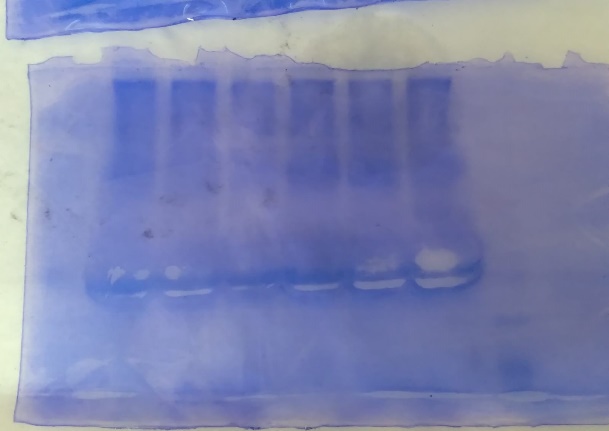


Selected bands

**ECC-1**

**
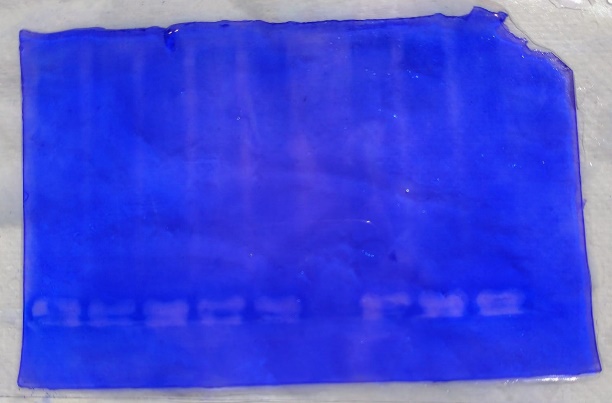

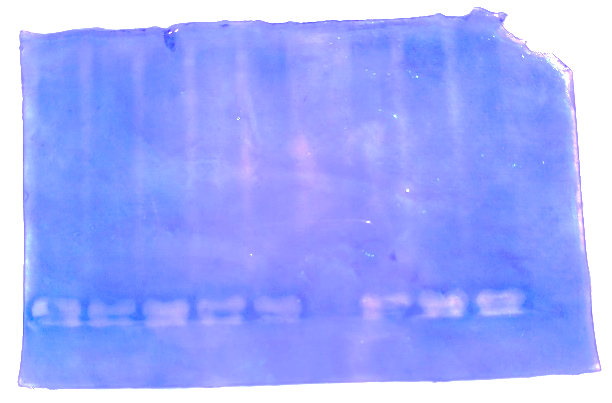
**Original image and converted to gray scale

Selected bands

**Figure 4.**

**ECC-1**

Antibody anti-N-cadherin


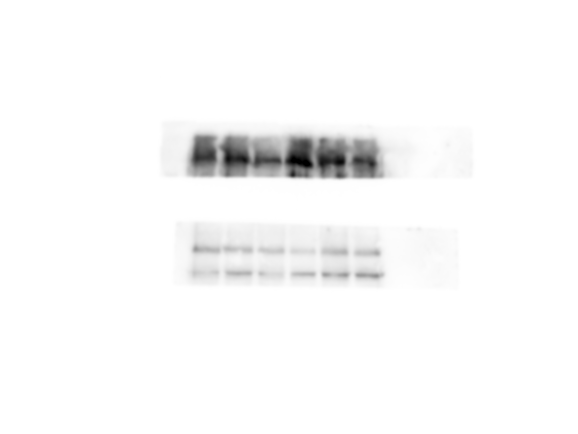
Original chemiluminescent image and converted to gray scale


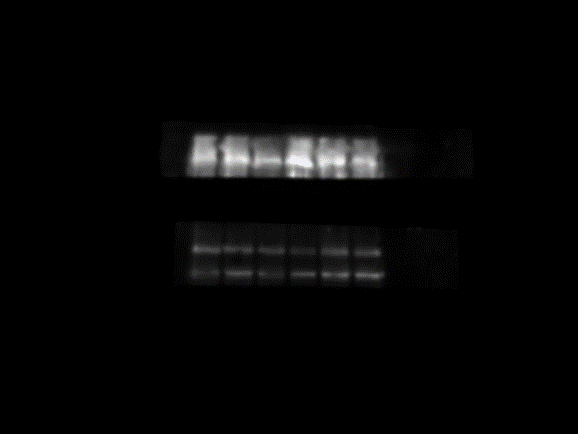


Selected bands

Antibody anti-E-cadherin

Original chemiluminescent image and converted to gray scale


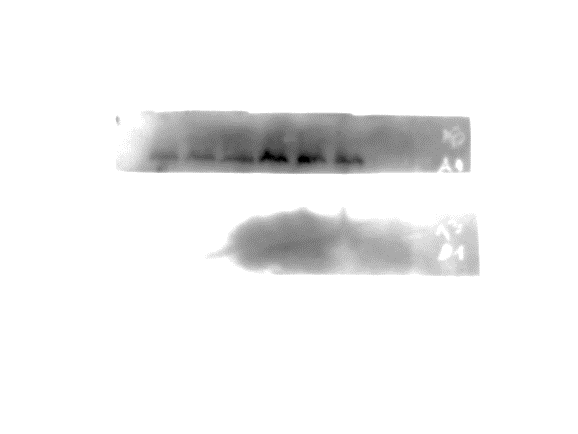


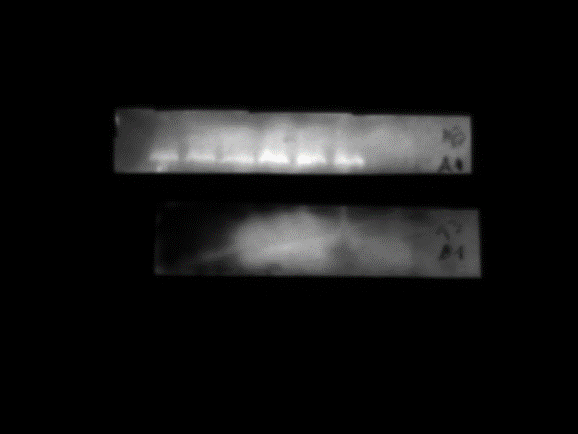
Selected bands

Antibody anti-pan-cadherin

Original chemiluminescent image and converted to gray scale


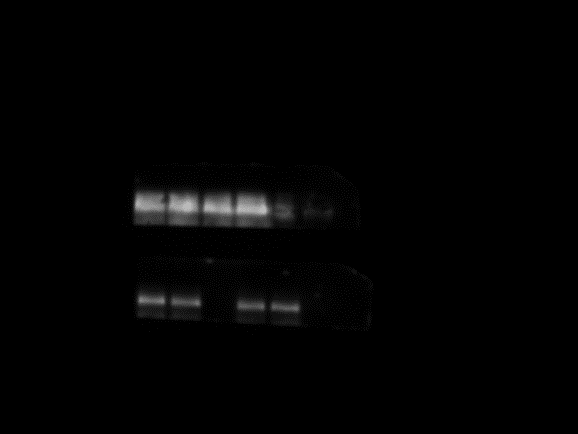

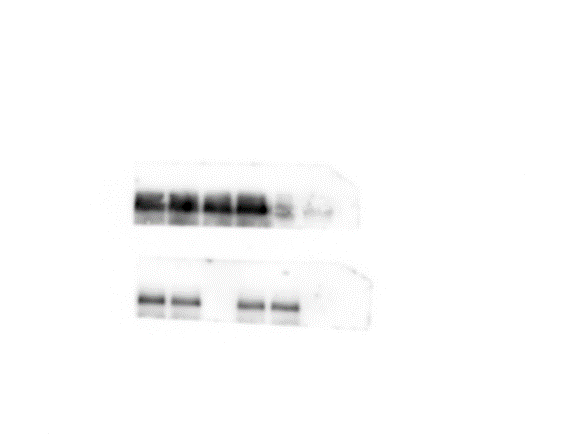


Selected bands

Antibody anti-β-catenin

Original chemiluminescent image and converted to gray scale


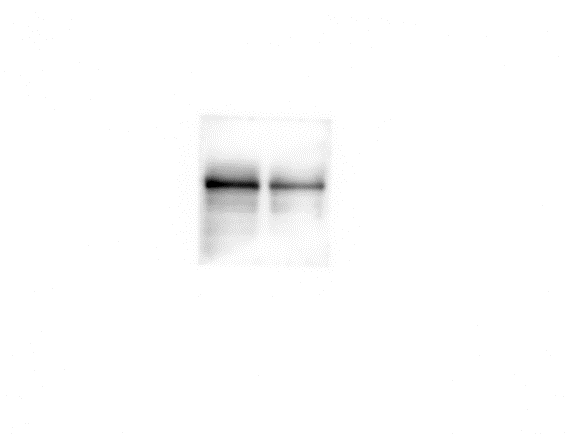

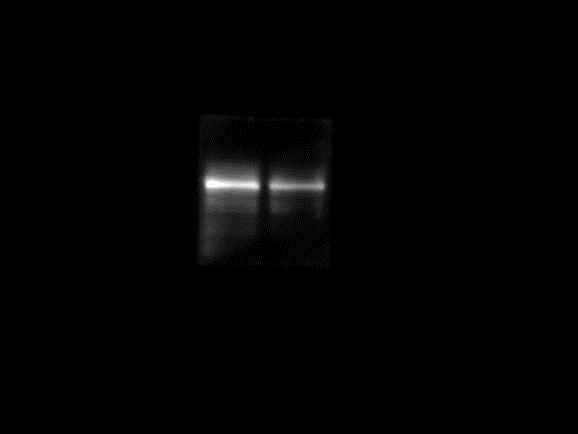


Selected bands

Antibody anti-β-actin

Original chemiluminescent image and converted to gray scale


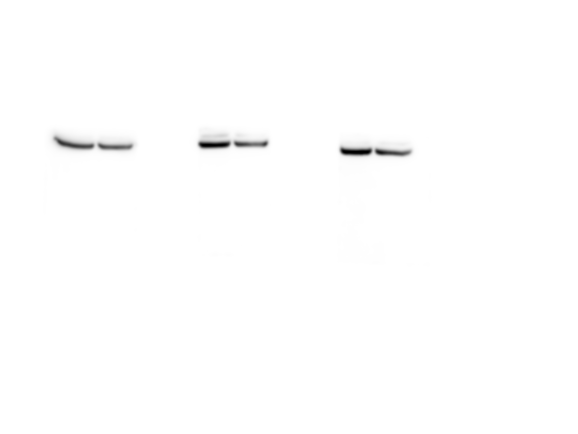


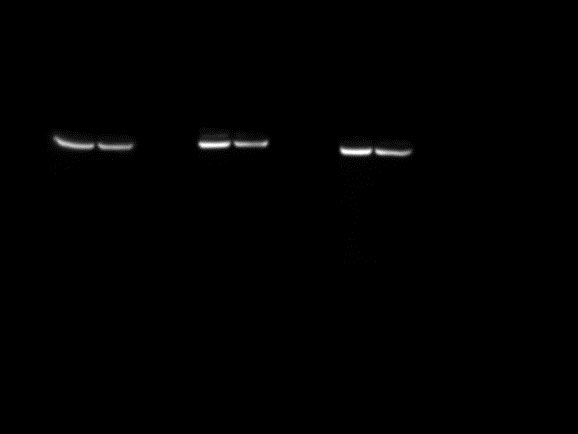


Selected bands

**HeLa**

Antibody anti-N-cadherin

Original chemiluminescent image and converted to gray scale


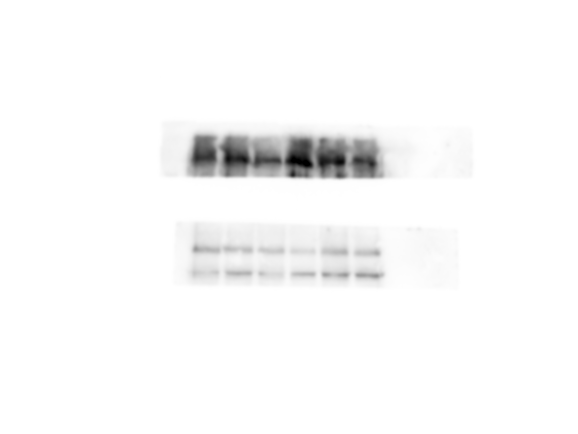

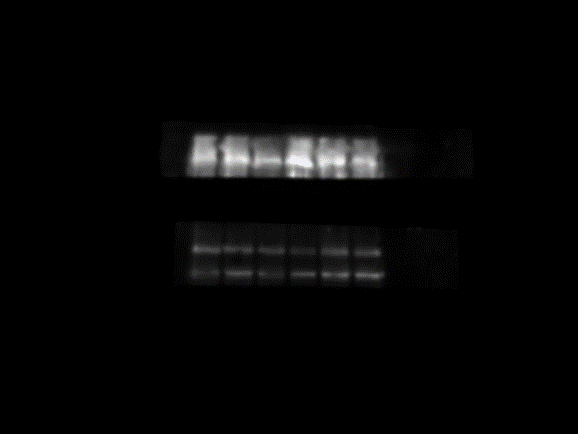


Selected bands

Antibody anti E-cadherin

Original chemiluminescent image and converted to gray scale


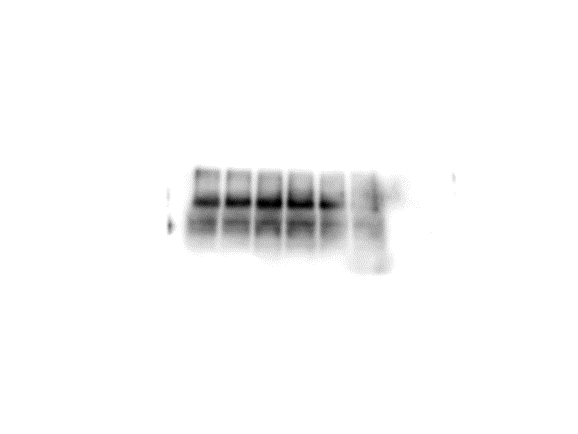


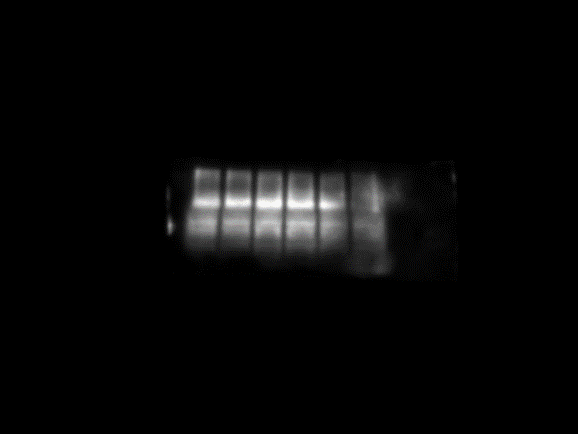


Selected bands

Antibody anti-pan-cadherin

Original chemiluminescent image and converted to gray scale


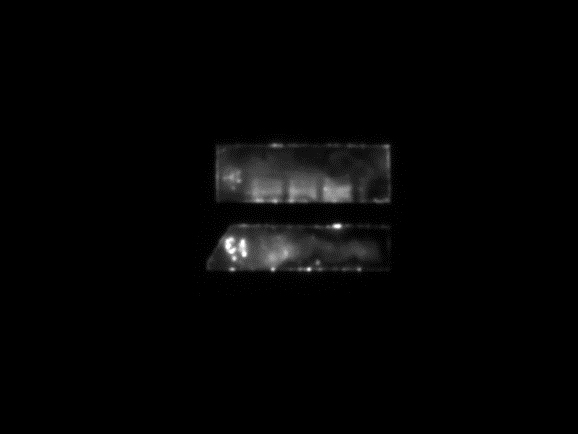

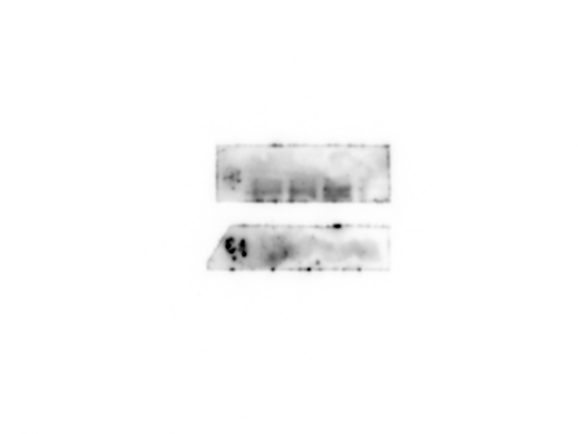


Selected bands

Antibody anti-β-catenin

Original chemiluminescent image and converted to gray scale


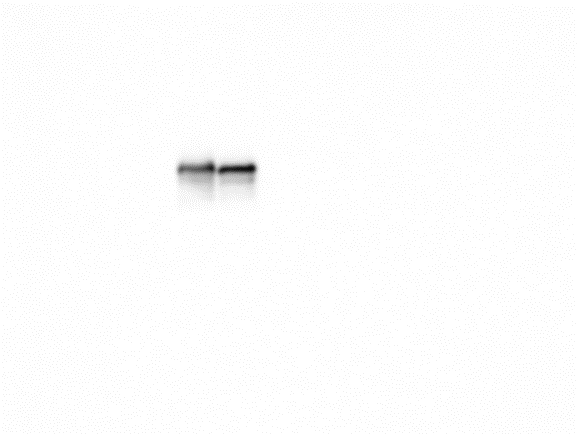

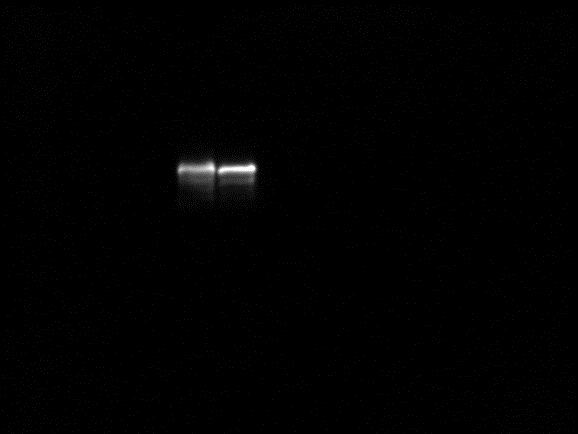


Selected bands

Antibody anti-β-actin

Original chemiluminescent image and converted to gray scale


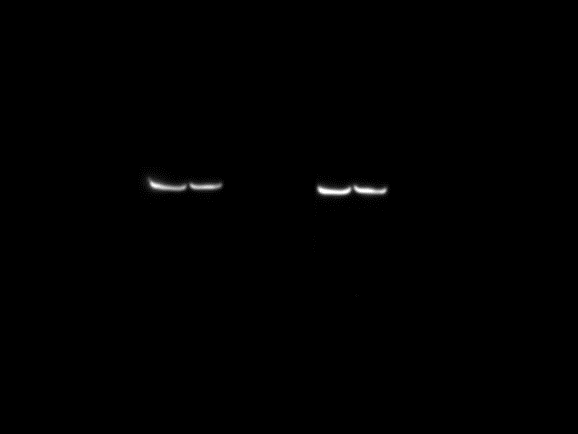

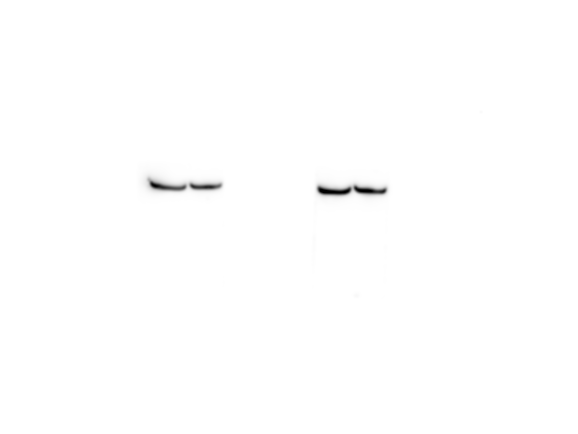


Selected bands

**Figure 5.**

**ECC-1**

Antibody anti-p-PDK1 (S241)

Original chemiluminescent image and converted to gray scale


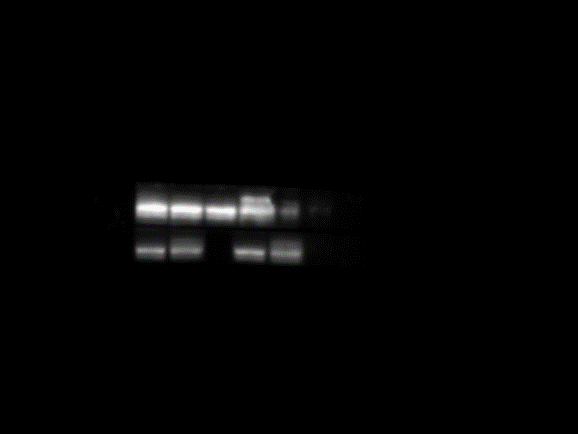

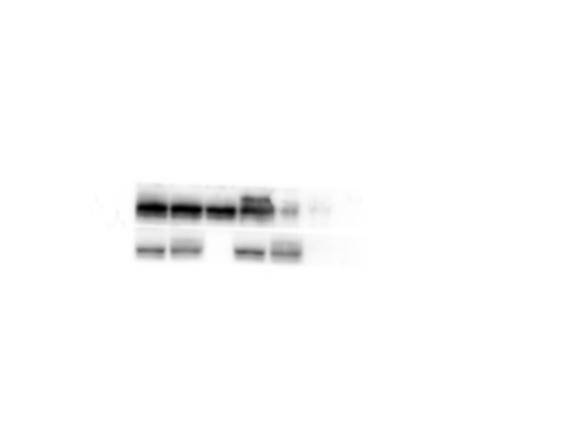


Selected bands

Antibody anti-p-Akt (S473)

Original chemiluminescent image and converted to gray scale


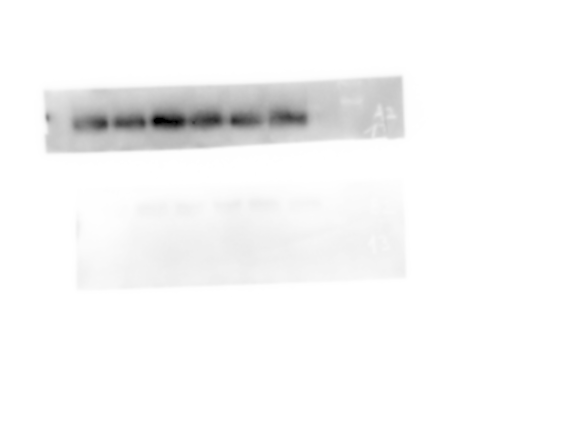


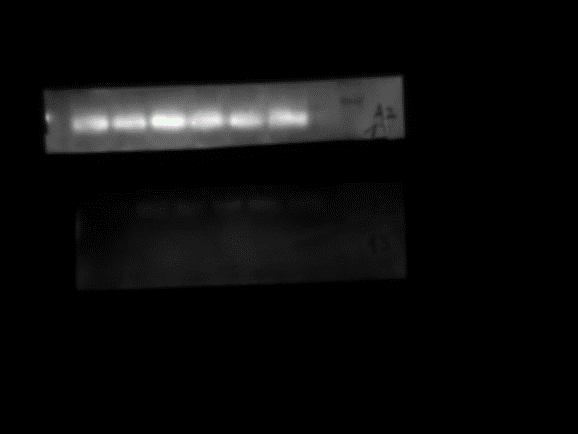


Selected bands

Antibody anti-p-Akt (T308)

Original chemiluminescent image and converted to gray scale


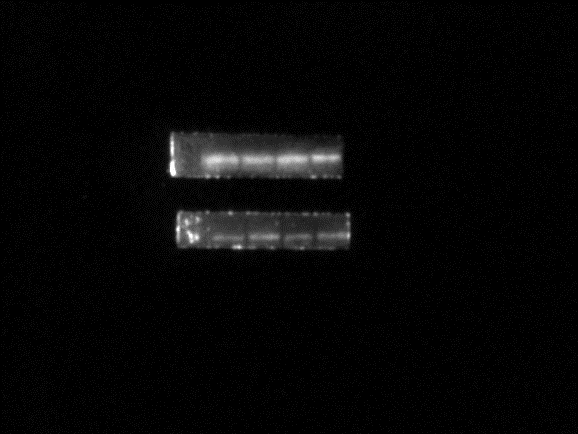

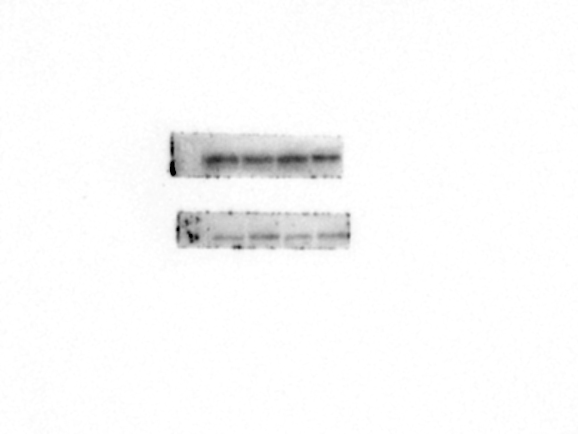


Selected bands

Antibody anti-pan-Akt

Original chemiluminescent image and converted to gray scale


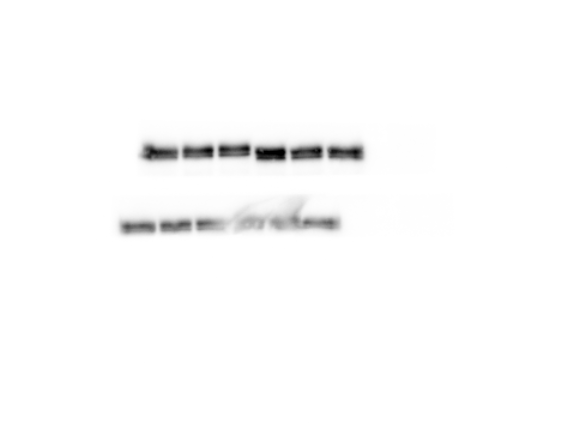


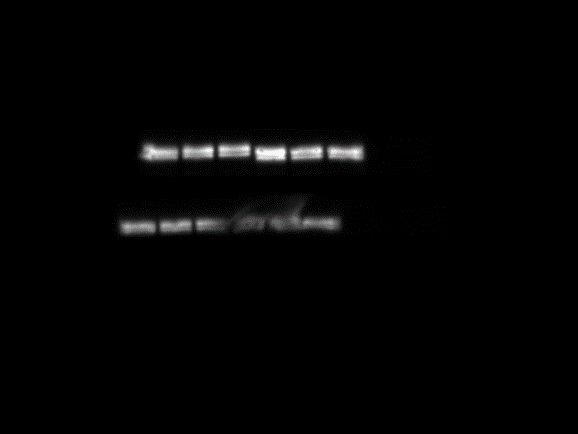


Selected bands

Antibody anti-PTEN

Original chemiluminescent image and converted to gray scale


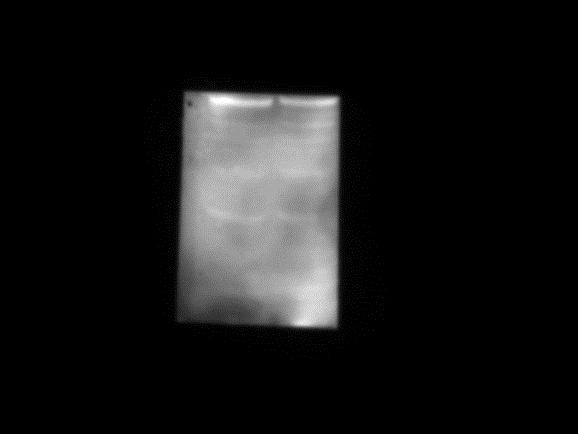

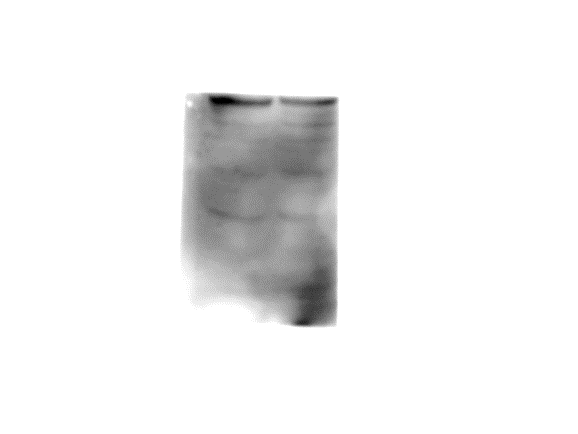


Selected bands

Antibody anti-p-PTEN

Original chemiluminescent image and converted to gray scale


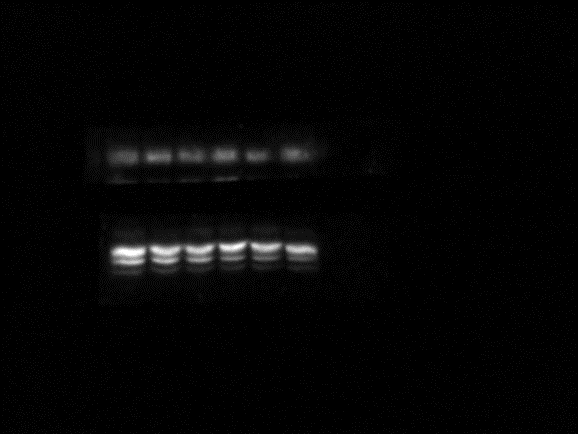

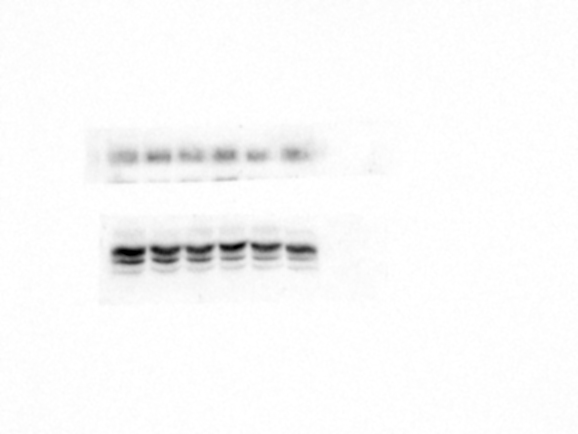


Selected bands

Antibody anti-p-GSK3β

Original chemiluminescent image and converted to gray scale


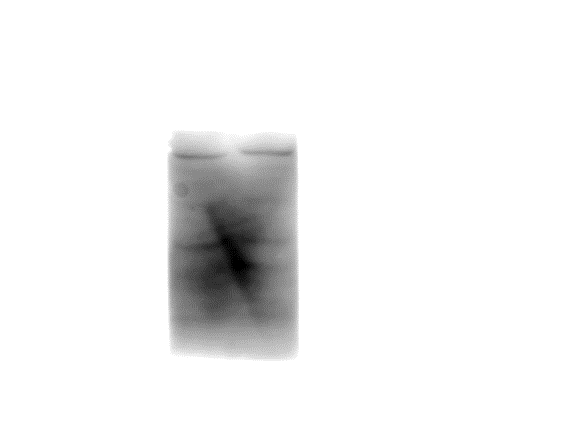


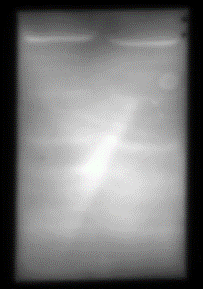


Selected bands

Antibody anti-p-c-Raf

Original chemiluminescent image and converted to gray scale


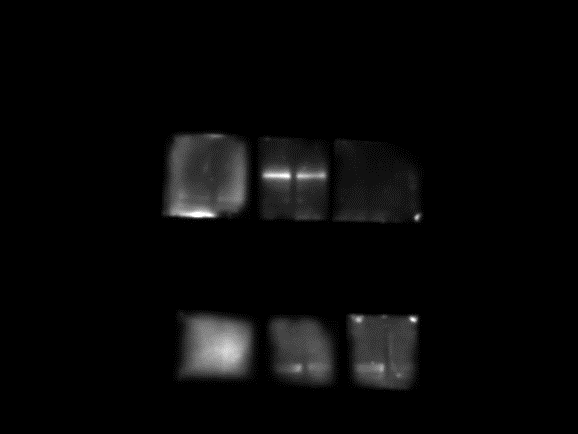

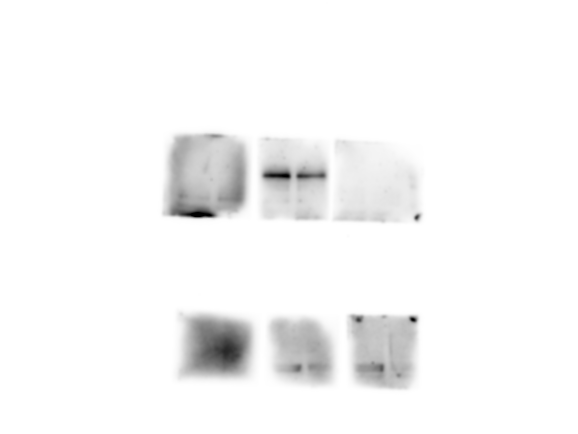


Selected bands

Antibody anti-β-actin

Original chemiluminescent image and converted to gray scale


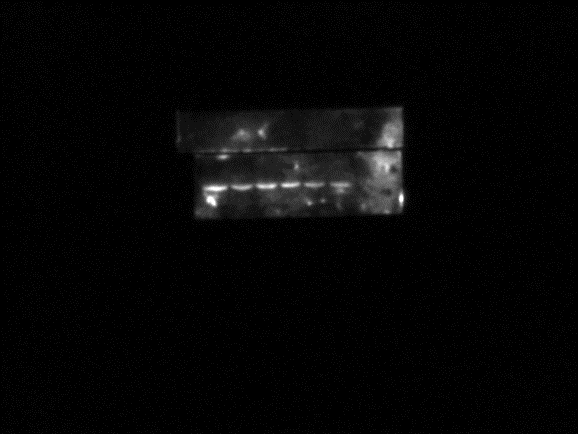

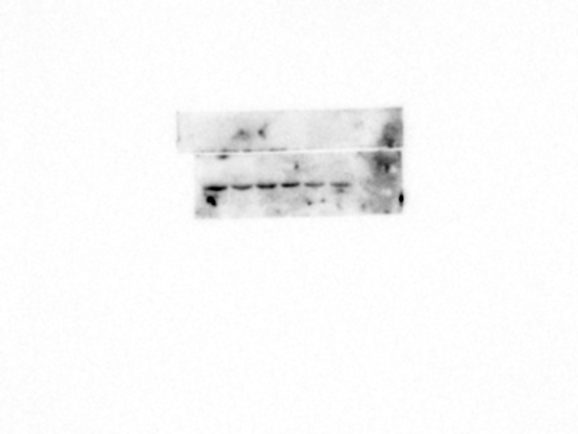


Selected bands

**HeLa**

Antibody anti-p-PDK1 (S241)

Original chemiluminescent image and converted to gray scale


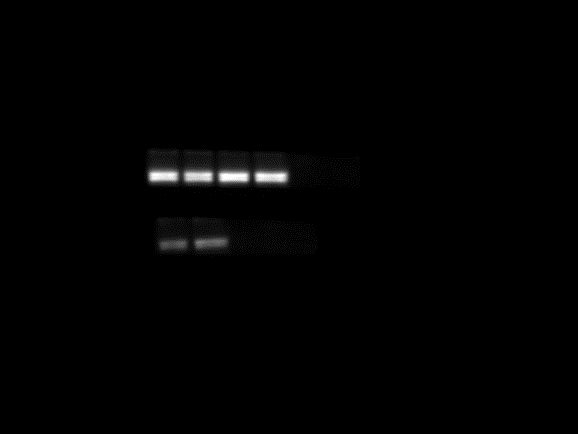

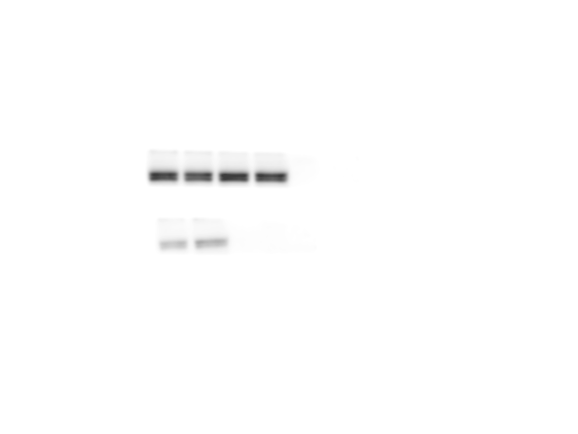


Selected bands

Antibody anti-p-Akt (S473)

Original chemiluminescent image and converted to gray scale


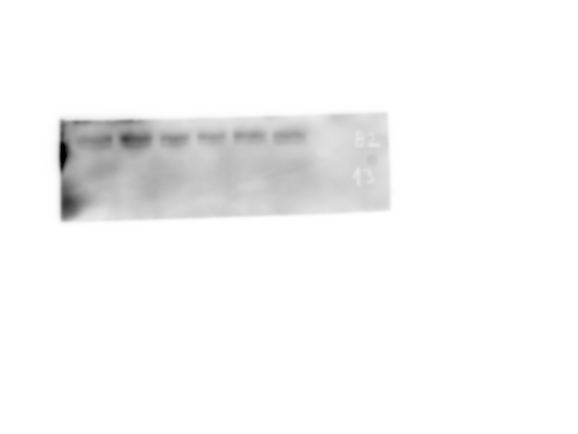


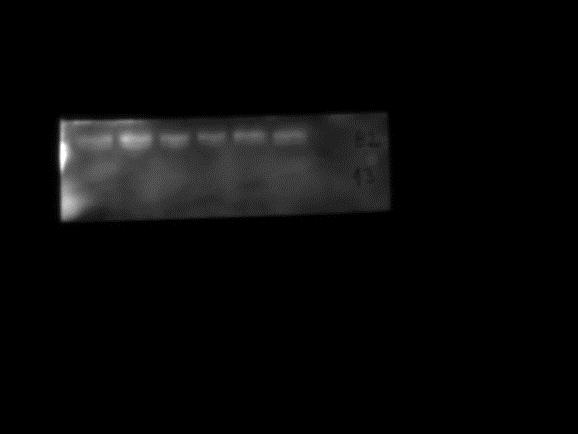


Antibody anti-p-Akt (T308)

Selected bands

Antibody anti-p-Akt (T308)

Original chemiluminescent image and converted to gray scale


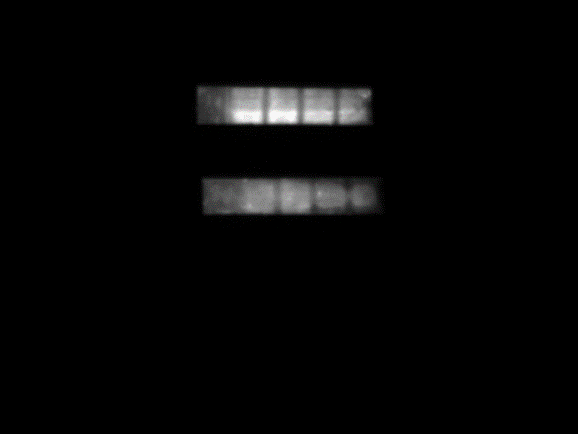

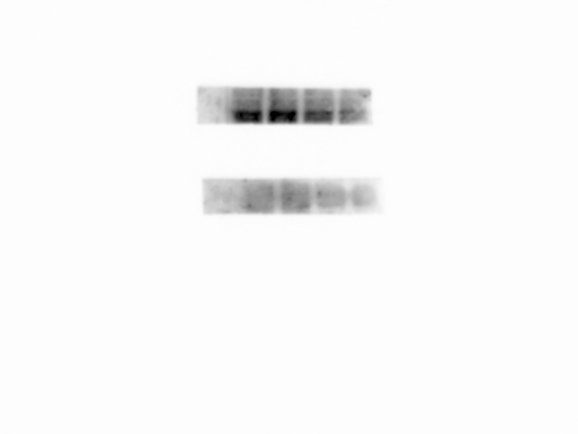


Selected bands

Antibody anti-pan-Akt

Original chemiluminescent image and converted to gray scale


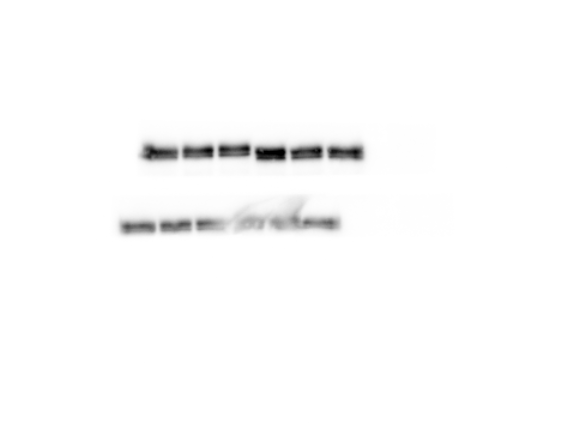


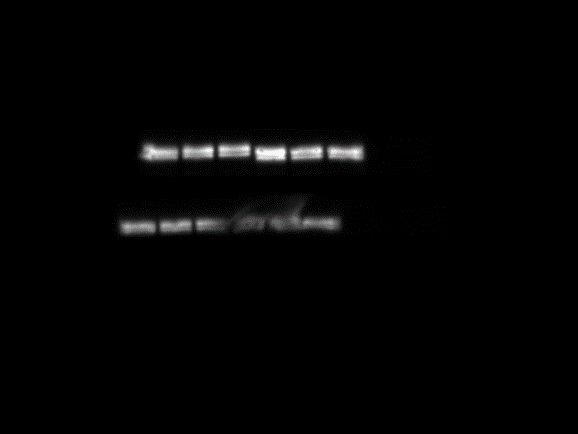


Selected bands

Antibody anti-p-PTEN

Original chemiluminescent image and converted to gray scale


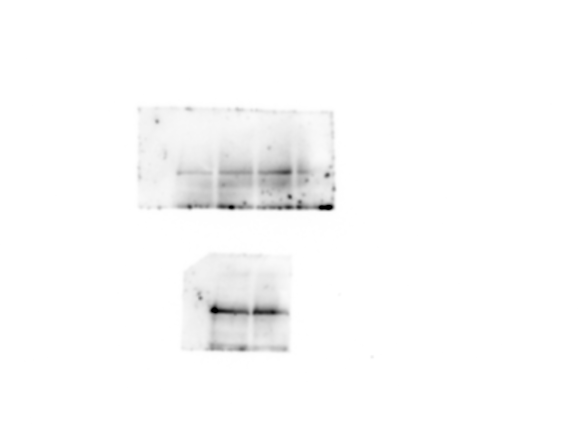


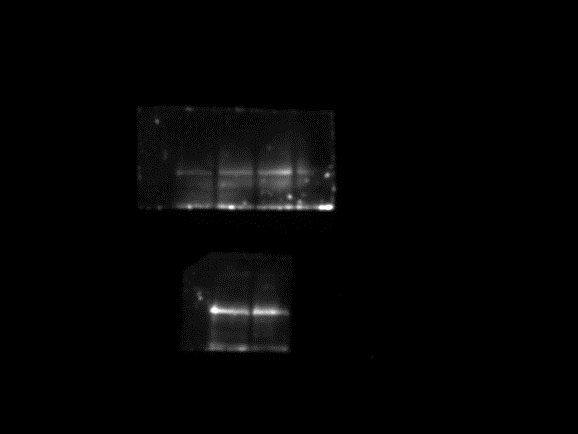


Selected bands

Antibody anti-PTEN

Original chemiluminescent image and converted to gray scale


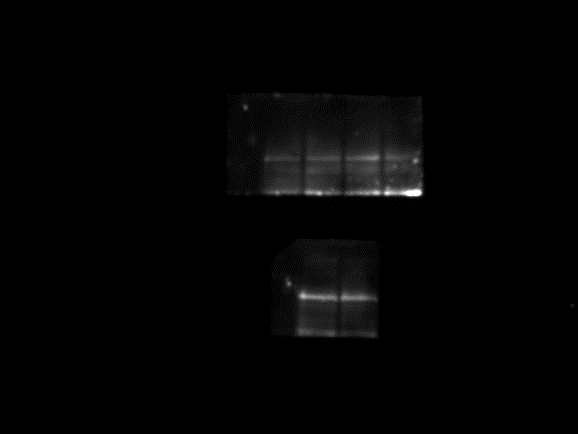

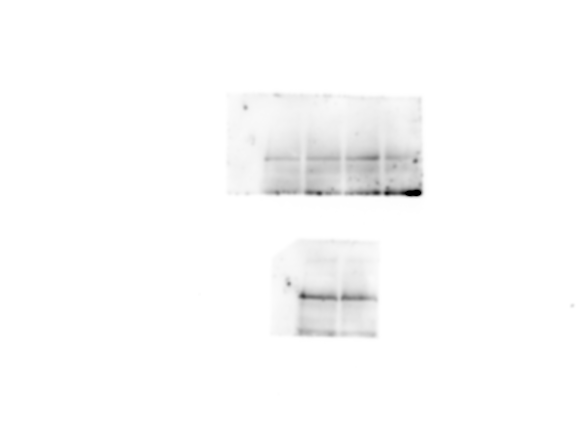


Selected bands

Antibody anti-p-GSK3β

Original chemiluminescent image and converted to gray scale


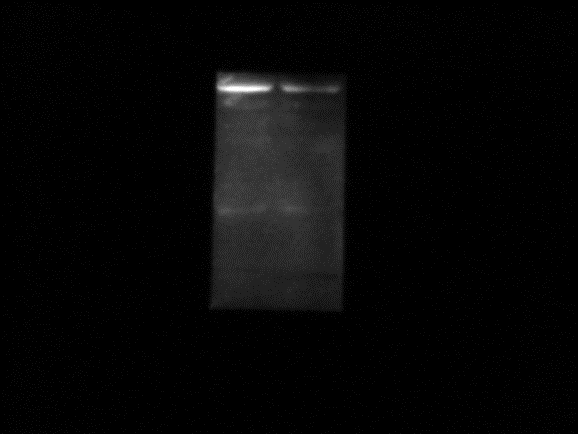

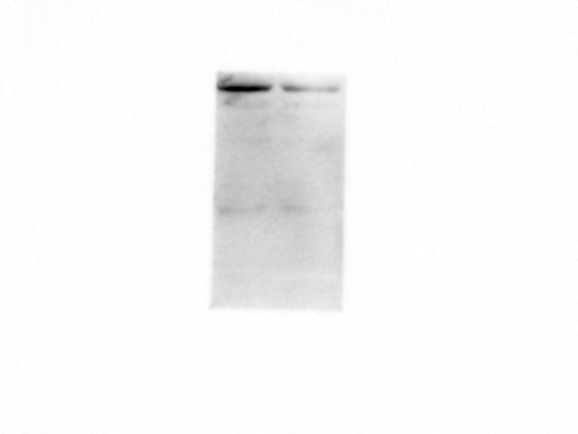


Selected bands

Antibody anti-p-c-Raf

Original chemiluminescent image and converted to gray scale


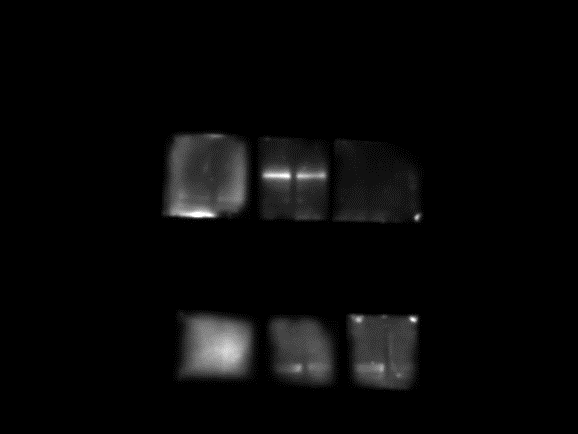

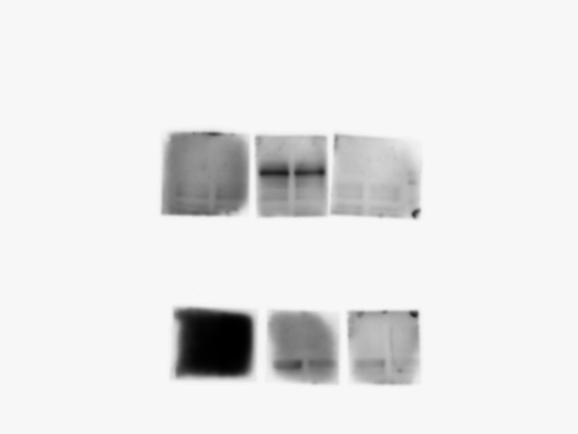


Selected bands

Antibody anti-β-actin

Original chemiluminescent image and converted to gray scale


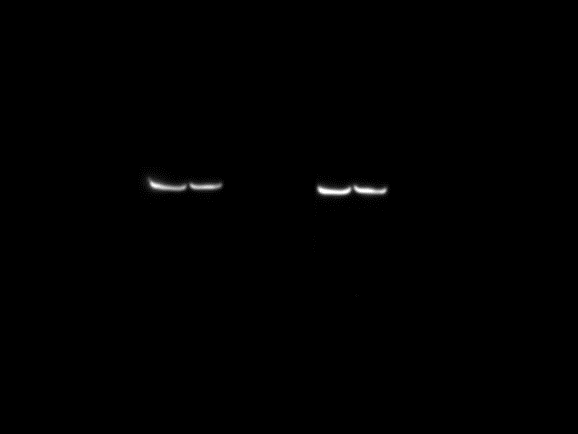

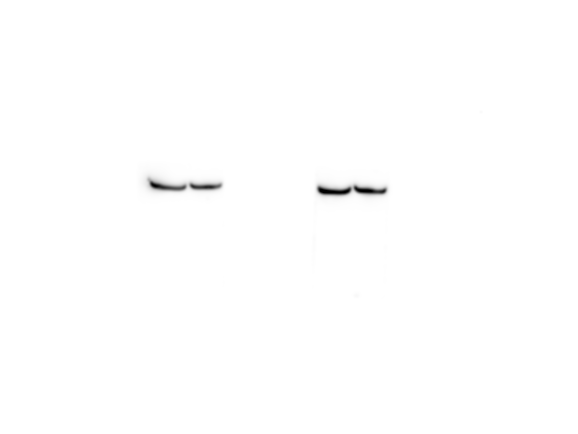


Selected bands
